# UNCOVERING THE DIMENSIONS OF WELL-BEING AMONG CAREGIVERS FOR OLDER AMERICANS IN THE NATIONAL STUDY OF CAREGIVING

**DOI:** 10.1093/geroni/igae098.2173

**Published:** 2024-12-31

**Authors:** Frances Yang, Maria Roche-Dean, Kristine Williams

**Affiliations:** University of Kansas Medical Center, Kansas City, Kansas, United States; University of Kansas, Kansas City, Kansas, United States; University of Kansas Medical Center, Kansas City, Kansas, United States

## Abstract

Evaluating caregiver well-being is vital for healthcare providers, researchers and policymakers. There are various valid and reliable instruments for caregiver well-being with as many as 45 items in Tebb’s Caregiver Well-Being Scale. When conducting survey research it is imperative to avoid participant burden and fatigue by limiting the number of questions. The National Study of Caregiving (NSOC) is a survey about caregivers’ responsibilities, health, and well-being while caring for their older care recipients. This study focuses on identifying and reducing the complex dimensions of the 17 well-being questions to yield a streamlined, valid, and reliable caregiver well-being measure in 2022. Exploratory factor analysis (χ2=1332, df=43, p<.001; RMSEA=0.1; CFI=0.96) and confirmatory factor analysis (χ2=1027, df=51, p<.001; RMSEA=0.09; CFI=0.97) were conducted with 2,376 caregivers’ responses in Round 12. Twelve questions were identified with high reliability (Cronbach’s alpha (α)=0.97) to best fit a three dimensional model for caregiver well-being: 1) Positive affect (cheerful mood, calm and peaceful, full of life; α=0.94), 2) Isolation (bored, lonely, life has meaning, life is lonely because I have few close friends; α=0.93), and 3) Psychological distress (upset, felt little interest, felt down depressed, felt nervous, felt unable to stop or control worrying; α=0.93). Reducing the number of valid caregiver well-being questions by 29% has the potential to decrease survey fatigue for caregivers. The dimensions or themes identified for caregivers’ responses about their well-being allows for appropriate development of targeted interventions and policy recommendations to improve informal caregiver well-being who are caring for those aging in the US.

